# Identification of Key Genes and Pathways in Persistent Hyperplastic Primary Vitreous of the Eye Using Bioinformatic Analysis

**DOI:** 10.3389/fmed.2021.690594

**Published:** 2021-08-13

**Authors:** Derin M. Thomas, Chitra Kannabiran, D. Balasubramanian

**Affiliations:** Kallam Anji Reddy Molecular Genetics Laboratory, Prof. Brien Holden Eye Research Center, LV Prasad Eye Institute, Hyderabad, India

**Keywords:** persistent hyperplastic primary vitreous, gene ontology, bioinformatic analysis, hub genes, molecular pathway

## Abstract

**Background:** The failure of the embryonic hyaloid vascular system to regress naturally causes persistent hyperplastic primary vitreous (PHPV), a congenital eye disease. PHPVs molecular pathway, candidate genes, and drug targets are unknown. The current paper describes a comprehensive analysis using bioinformatics to identify the key genes and molecular pathways associated with PHPV, and to evaluate potential therapeutic agents for disease management.

**Methods:** The genes associated with PHPV were identified using the pubmed2ensembl text mining platform. GeneCodis was employed to evaluate the Gene Ontology (GO) biological process terms and Kyoto Encyclopedia of Genes and Genomes (KEGG) pathways. Search Tool for the Retrieval of Interacting Genes (STRING) constructed a protein-protein interaction (PPI) network from the text mining genes (TMGs) in Cytoscape. The significant modules were clustered using Molecular Complex Detection (MCODE), and the GO and KEGG analysis for the hub genes were analyzed with the Database of Annotation, Visualization and Integrated Discovery (DAVID) tool. ClueGO, CluePedia, and ShinyGo were used to illustrate the functions and pathways of the clustered hub genes in a significant module. The Drug-Gene Interaction database (DGIdb) was used to evaluate drug–gene interactions of the hub genes to identify potential PHPV drug candidates.

**Results:** A total of 50 genes associated with PHPV were identified. Overall, 35 enriched GO terms and 15 KEGG pathways were discovered by the gene functional enrichment analysis. Two gene modules were obtained from the PPI network constructed with 31 nodes with 42 edges using MCODE. We selected 14 hub genes as core candidate genes: *TP53, VEGFA, SMAD2, CDKN2A, FOXC, FZD4, LRP5, KDR, FZD5, PAX6, MYCN, NDP, PITX2*, and *PAX2*, primarily associated with camera-type eye morphogenesis, pancreatic cancer, the apoptotic process involved in morphogenesis, and the VEGF receptor signaling pathway. We discovered that 26 Food and Drug Administration (FDA)-approved drugs could target 7 of the 14 hub genes.

**Conclusions:** In conclusion, the results revealed a total of 14 potential genes, 4 major pathways, 7 drug gene targets, and 26 candidate drugs that could provide the basis of novel targeted therapies for targeted treatment and management of PHPV.

## Graphical Abstract

Schematic diagram for evaluating and identifying the key genes, molecular pathways, predicted drug targets of PHPV. 
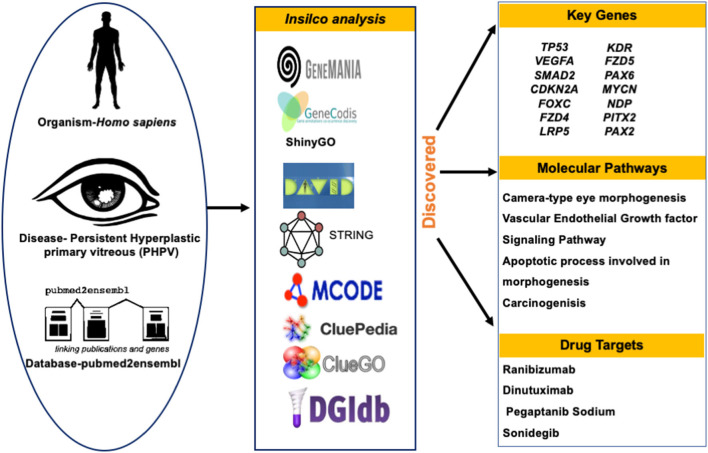

## Introduction

**Figure 1:**
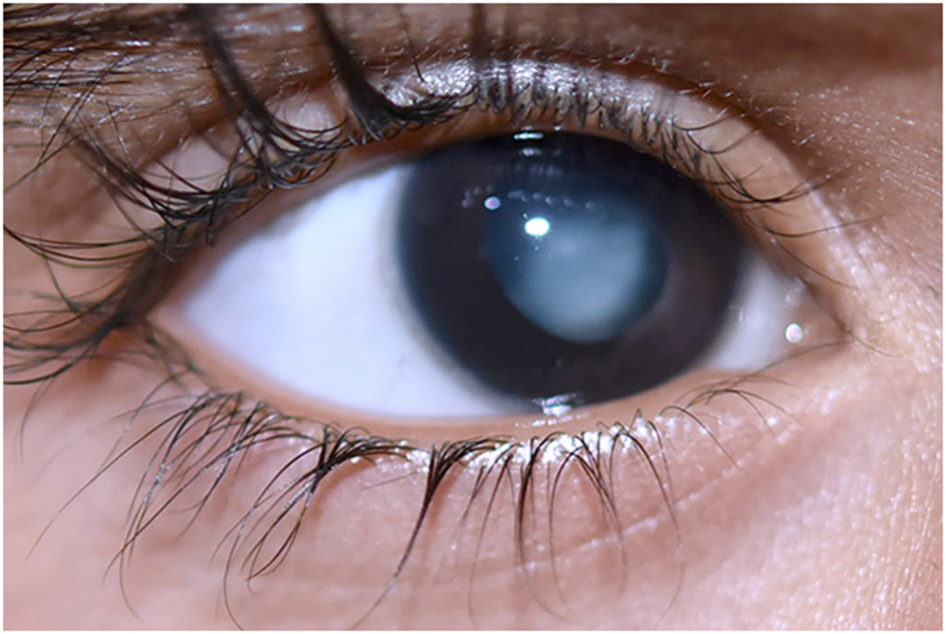
Morphology of persistent hyperplastic primary vitreous in a 1-year-old boy with white vascularized retrolental tissue. Image obtained with prior informed consent from the parents of the patient.

Persistent hyperplastic primary vitreous (PHPV) is a rare vitreoretinal disorder which accounts for up to 5% of blindness (1). PHPV's pathophysiology occurs during the embryonic stage, with vessel development occurring in the third week of pregnancy. The hyaloid artery system expands and extends to the anterior part of the eye forming the iridohyaloid or capsulopupillary artery during this period. At this point of development, the posterior tunica vasculosa lentis, which is an anastomosis of vessels at the back of the lens begins to develop and nourishes the lens. Secondary vitreous begins to develop in place of primary vitreous during the second trimester of pregnancy. The pathological persistence of fetal intraocular vessels including the hyaloid artery in embryonic vitreous causes this congenital eye disease (2, 3). Apoptosis or macrophage activation causes hyaloidal artery regression which is accompanied by vasa hyaloidal propria, iridohyaloid, and tunica vasculosa lentis (4). White retrolental tissue, an anteriorly swollen lens, centrally dragging ciliary structures, and varying degrees of lenticular opacification are the most prominent clinical symptoms (5). These vascular remnants can hinder the normal retinal development leading to retinal detachment and optic nerve or macula anomalies, and it can also appear in anterior, lateral, or combined forms in various patients (5, 6). PHPV is usually detected in infants within the first 3 months of life due to leukocoria, microphthalmos, and strabismus (7). Figure 1 represents the typical morphology of a PHPV subject. PHPV is also known as persistent fetal vasculature (PFV) (5). Bilateral PHPV is rare and sporadic compared to unilateral PHPV; however, it is an autosomal dominant or recessive trait that may be inherited (8, 9).

A large number of genes are involved in the development and regression of the hyaloid artery. PHPV traits have been observed in human and animal models. In humans, PHPV incidence was found to be an autosomal dominant inheritance pattern in an Egyptian family (10). Mutations in the *NDP* gene and the *COX15* gene on chromosome 10 have been found in cases of bilateral PHPV (11–14). The *ZNF408* gene, which had previously been found in retinitis pigmentosa and autosomal dominant familial exudative vitreoretinopathy (ADFEVR) was also identified in PHPV cases of microcornea, posterior megalolenticonus, and coloboma syndrome (MPPC syndrome) (15). *FZD4* (frizzled-type receptor 4) was reported to be associated in certain PHPV cases as well as a gene linked to familial exudative vitreoretinopathy (FEVR) (16). In animal models, various signaling pathways have been implicated in the pathogenesis of PHPV including protooncogene *ski, p53*, tumor suppressor gene *Arf*, ephrin-B2, βA3/A1-crystallin, *LRP5, ang-2, Bax* and *Bak, FZD4*, and *ephrin-A5*. FEVR, incontinentia pigmenti, retinoblastoma, and retinopathy of prematurity (RoP) are some of the conditions that mimic PHPV-like symptoms (17–23). However, the regulatory mechanisms responsible and genes involved in the process of fetal vascular regression continue to be unclear, as does the underlying cause of failure of regression.

The current surgical management of PHPV is primarily based on the pathological presence of individual cases. Depending on the ocular pathology of PHPV, the limbal and pars plicata incisions are the two most frequent surgical incision methods (24). The most common criteria for surgical intervention are severe media opacities due to cataract or retrolental membranes, progressive anterior chamber shallowing due to cataract, uncontrolled glaucoma or secondary ocular hypotony related to ciliary process dragging, vitreous hemorrhage, and retinal detachment following vitreoretinal traction (5, 25). In cases with advanced pathology, such as acute optic nerve hypoplasia, severe retinal detachment, or microphthalmia, surgery is not a preferred choice since post-operative vision is often low (24). Non-surgical management is currently used in non-progressive conditions and patients with non-central opacity that does not cause any visual impairment. If a non-surgical alternative is used, diligent follow-up should be carried out to detect any potential risks, such as cataract progression or glaucoma (26). The disease's heterogeneity continues to render PHPV diagnosis and treatment challenging.

Since PHPV is a rare disease, understanding the mechanisms that constitute a group of phenotypes is often restricted by small sampling sizes. Therefore, comprehending the molecular mechanisms underlying the expression of the mutated gene, which leads to improper vascular remodeling and the formation of PHPV is often individual-specific and critical for diagnosis, prevention, and therapeutic management. The assessment and analysis of molecular pathways and genetic variant analysis using conventional variant detection approaches such as Sanger sequencing, next generation sequencing, FISH, aCGH, and GTG banding can be time-consuming, expensive, and results in complicated data analysis for unspecified variants (27–31). Text mining is an effective tool for generating a hypothesis since it can reveal novel correlations between genes and the disease pathologies (32). Integration of text mining with biological knowledge and a bioinformatic approach provides new insights into the potential to reconfigure existing drugs (33). By integrating biological databases and *in silico* tools, the present paper aims to explore possible molecular mechanisms (if any) and classify the causative genes responsible for the heterogenic disease PHPV, thus discovering new drug targets for the treatment of the disease.

## Methods

### Selection of Key Genes Using Text Mining Analysis

**Figure 2:**
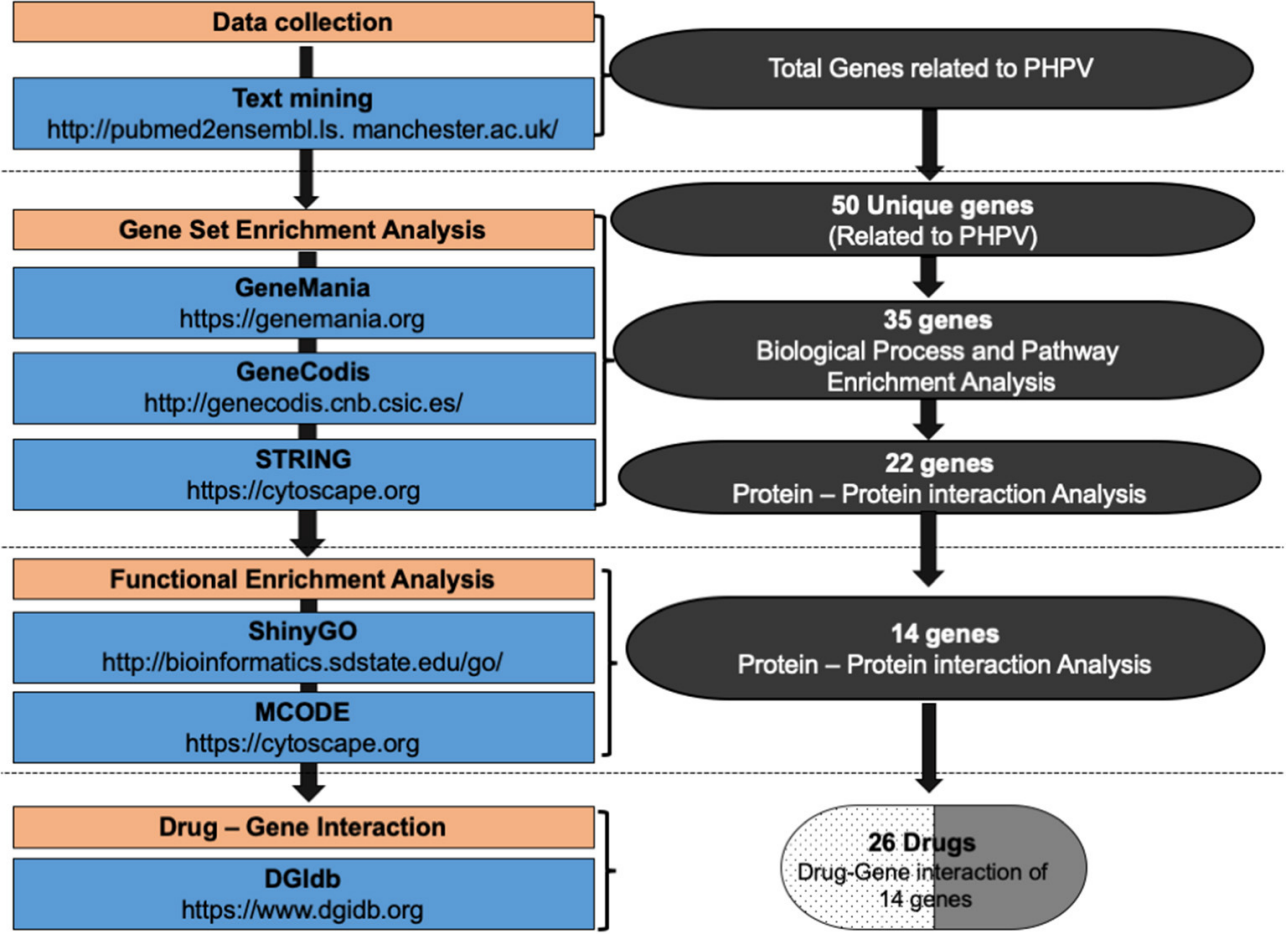
Summary of the study design and methodology flowchart. Text mining was conducted using pubmed2ensembl to identify genes associated with persistent hyperplastic primary vitreous (PHPV). GeneCodis was used to detect genes enriched in Gene Ontology (GO) biological process terms and KEGG pathways. STRING and MCODE software were used to construct a protein-protein interaction network and identify modules. The GO biological process terms and KEGG pathways were analyzed using DAVID, ClueGO, and ShinyGo. The drug list was compiled based on the gene-drug interaction using the drug-gene interaction database (DGIdb).

To identify genes related to PHPV, text mining analysis was performed using pubmed2ensembl (http://pubmed2ensembl.ls.manchester.ac.uk) which revealed associations between genes and the literature for data extraction. It is a freely accessible database that connects over 2,000,000 articles in PubMed publications to 150,000 Ensembl genes from 50 species (34, 35). To create a list of key genes, we used search terminology “Persistent Hyperplastic Primary Vitreous” and “Persistent Fetal Vasculature” from 100,000 relevant document IDs. The search terms used were confined to avoid overlapping genes related with other ocular disorders. The species dataset was set to “*Homo sapiens* (GRCh37)” and the query result was constrained using “filter on MEDLINE: PubMed ID”. The unduplicated genes were extracted and the TMGs were recovered as the intersection of gene hits from the two sets. Figure 2 represents the methodology flowchart and summary of the study design.

### Pathway Enrichment and Biological Process Analysis

The TMGs obtained from text mining were analyzed for biological process annotations. The tool GeneCodis, a web-based server, was used (http://genecodis.cnb.csic.es/) to execute an enrichment analysis of the TMGs. GeneCodis assesses functional analysis of gene lists that integrates different sources of information which includes Gene Ontology (GO) [a collection of terminology that describe gene products in terms of Biological Process (BP), Molecular Function (MF), and Cellular Component (CC)], KEGG pathways (offers evidence on biological metabolic pathways that are well-known), and Inter Pro motifs (36). The organism chosen for the analysis was set as *Homo sapiens*. The TMGs were used as the input set, and genes with significantly enriched biological processes relevant to eye development and vasculogenesis were chosen using an adjusted *P*-value and analyzed using the GO and BP categories. Using the same method, the genes from the selected annotations were used for KEGG pathway analysis and the genes obtained by the KEGG pathway analysis were further analyzed (28). GeneMania (version 3.5.2), a Cytoscape plugin (version 3.8.2), was used to construct a gene-gene functional interaction network from the TMGs. The advanced statistical options used were max resultant genes = 20, max resultant attributes = 10, and the automatically selected network weighting function. The resulting network comprised functional annotations from GO as well as genes most closely related to the original list.

### Construction of Protein-Protein Interaction Network and Module Analysis

STRING (version 1.6.0) was used to construct the PPI network of 35 enriched genes based on GO. STRING is a web-based database comprising nearly 24.6 million proteins and over 3.1 billion interactions from 5,090 distinct species [https://string-db.org/cgi/input.pl; (37)]. The fundamental metrics of nodes in network theory are connectivity degree (k), Betweenness Centrality (BC), Closeness Centrality (CC), Eigenvector Centrality (EC), and eccentricity. However, the main advantage of PPI network analysis is to accommodate a wide range of biological processes including inputs pathway information, providing confidence scores based on evidence from conserved genomic neighborhoods, gene-fusion events, co-occurrence events, co-expression data, experimental data, database information, text mining, and homology. In the PPI network, nodes with a high degree known, as hub proteins, are critical proteins because they may correlate to disease-causing genes while nodes with a high BC, known as bottlenecks, prefer to signify important genes because they can be compared to highly used intersections on major highways or bridges. The confidence score of 0.900 was specified as the minimum criterion. The molecular interaction network was then visualized and hub genes were identified using the Cytoscape software which visually presents the integration of gene expression, biological network, and genotype (38). In this study, the hub nodes were classified by a high score based on the network's scale-free property and was used for centrality analysis by analyzing the network topology (39) and considered the sub-network of these key proteins as the backbone which was worth exploring further in the signaling pathways involved in eye development. Further, a built in Cytoscape plugin Molecular Complex Detection (MCODE, version 2.0.0) was used to distinguish the significant gene modules (clusters) and hub genes from the PPI network (40). The cutoff parameters were “degree cutoff = 2,” “node score cutoff = 0.2,” “k-core = 2,” and “max depth = 100” (41).

### Drug-Gene Interactions

The Drug-Gene Interaction Database (DGIdb) (www.dgidb.org) is an online resource that consolidates data from various sources to illustrate drug–gene interactions and gene druggability (42). We investigated drug-gene interactions used in significant module genes as the potential targets for existing drugs or compounds using DGIdb (Version 3.0). The PubChem database was used to obtain the chemical structure of the identified drugs (https://pubchem.ncbi.nlm.nih.gov). It has over 25 million specific chemical structures and 90 million bioactivity outcomes linked to thousands of macromolecular targets.

## Results

### Identification of Candidate Genes

**Figure 3:**
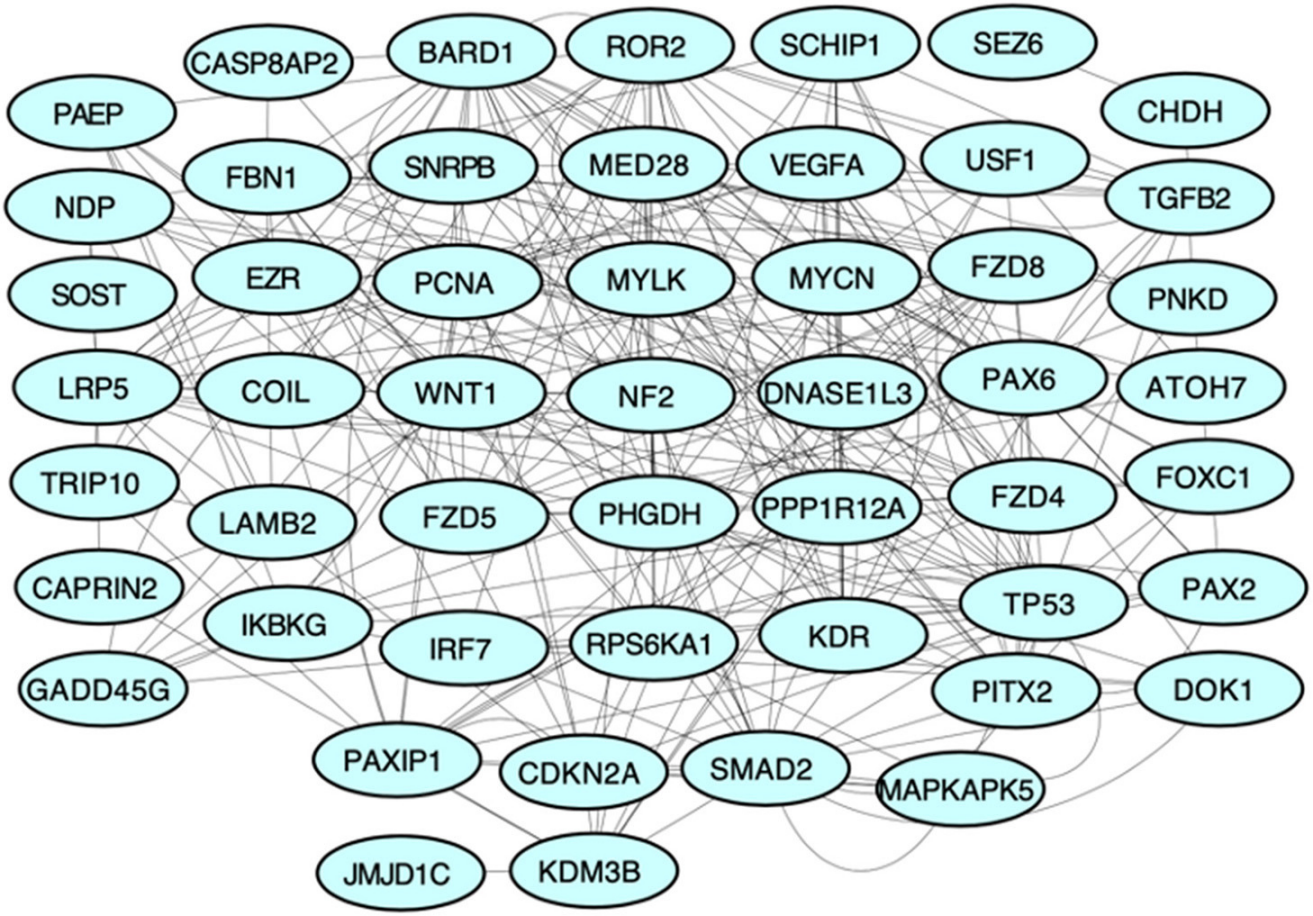
Protein-protein interaction network of all TMGs related to PHPV. The network, genetic interactions, co-expression analysis, and pathways evaluated using Genemania, a Cytoscape plugin, are represented in the figure.

We obtained 50 unique genes in *Homo sapiens* associated with PHPV using the TMG approach. Figure 3 depicts the network, genetic interactions, co-expression analysis, and pathways of the 50 TMGs assessed by GeneMania. From these, 35 genes were selected as candidate genes for enrichment analysis based on their GO and molecular pathways.

### Enrichment Analysis of TMGs

The most enriched terminology directly linked to the pathology of vasculature morphogenesis of the eye contributing to PHPV was identified using GeneCodis (with *P* = 1.00E-07), GO, biological process (BP), and KEGG. The GO and BP annotations analysis identified 35 significantly enriched genes. The 10 most enriched functions were “extracellular matrix cell signaling” (*P* = 2.33E-08), “multicellular organism development” (*P* = 2.51E-08), “regulation of transcription by RNA polymerase ii” (*P* = 3.12 E-08), “Norrin signaling pathway” (*P* = 3.50E-08), “regulation of transcription, DNA templated” (*P* = 3.77E-08), “negative regulation of gene expression” (*P* = 1.27E-07), “retina vasculature morphogenesis in camera-type eye” (*P* = 1.53E-07), “heart development” (*P* = 2.04E-06), “vascular endothelial growth factor (VEGF) signaling pathway” (*P* = 2.17E-06), and “post-embryonic camera type eye development” (*P* = 4.52E-05). Overall, 15 major pathways involving 12 TMGs were discovered by KEGG enrichment analysis. The five most significantly enriched pathways were “pathways in cancer” (*P* = 11.24E-12), “proteoglycans in cancer” (*P* = 5.54E-12), “hepatocellular carcinoma” (*P* = 3.16E-11), “gastric cancer” (*P* = 3.94E-11), and “pancreatic cancer” (*P* = 8.69E-10), involving 9, 7, 6, 6, and 5 text mining genes, respectively. Table 1 displays 15 enriched GO terms and Table 2 exhibits the KEGG analysis of 10 enriched molecular pathways of the TMGs.

**Table 1:** Top 15 enriched Gene Ontology (GO) biological process terms assigned to the text mining genes.

**Biological process**	**Genes in query set**	**Total genes in the genome**	**Corrected *P*-value**	**Genes**
Extracellular matrix-cell signaling	3	4	2.33E-08	*FZD4, NDP, LRP5*
Multicellular organism development	9	1,103	2.51E-08	*FZD4, FZD5, VEGFA, PAX2, PAEP, LRP5, KDR, ATOH7, FOXC1*
Regulation of transcription by RNA polymerase ii	9	1,102	3.12E-08	*VEGFA, TP53, PAX2, MYCN, SMAD2, JMJD1C, ATOH7, FOXC1, KDM3B*
Norrin signaling pathway	3	3	3.50E-08	*FZD4, NDP, LRP5*
Positive regulation of transcription, DNA-templated	8	701	3.77E-08	*FZD4, TP53, PAX2, NDP, MYCN, SMAD2, LRP5, FOXC1*
Positive regulation of transcription by RNA polymerase ii	9	1,068	4.73E-08	*IKBKG, FZD5, VEGFA, TP53, PAX2, MYCN, SMAD2, LRP5, FOXC1*
Negative regulation of gene expression	6	291	1.27E-07	*VEGFA, TP53, TGFB2, MYCN, SMAD2, KDR*
Retina vasculature morphogenesis in camera-type eye	3	7	1.53E-07	*FZD4, NDP, LRP5*
Heart development	5	234	2.04E-06	*TP53, TGFB2, PCNA, SMAD2, FOXC1*
Vascular endothelial growth factor signaling pathway	3	16	2.17E-06	*VEGFA, KDR, FOXC1*
Regulation of transcription, DNA-templated	7	998	4.75E-06	*TP53, PAX2, MYCN, SMAD2, JMJD1C, ATOH7, FOXC1*
Negative regulation of cell population proliferation	5	438	3.78E-05	*FZD5, TP53, TGFB2, NF2, SMAD2*
Positive regulation of epithelial to mesenchymal transition	3	48	4.27E-05	*TGFB2, SMAD2, FOXC1*
Post-embryonic camera-type eye development	2	4	4.52E-05	*FZD5, VEGFA*
Heart morphogenesis	3	53	4.76E-05	*VEGFA, TGFB2, FOXC1*
*In utero* embryonic development	4	202	4.76E-05	*VEGFA, TP53, SMAD2, FOXC1*
Positive regulation of endothelial cell chemotaxis by VEGF-activated vascular endothelial growth factor receptor signaling pathway	2	5	4.90E-05	*VEGFA, KDR*
Vascular endothelial growth factor receptor-2 signaling pathway	2	5	4.90E-05	*VEGFA, KDR*
Positive regulation of cell population proliferation	5	519	4.96E-05	*VEGFA, TGFB2, PAX2, LRP5, KDR*
Cell differentiation	6	943	5.02E-05	*VEGFA, PAX2, SMAD2, KDR, ATOH7, FOXC1*
Positive regulation of gene expression	5	486	5.03E-05	*VEGFA, TP53, MYCN, SMAD2, FOXC1*
Vasculogenesis	3	61	5.39E-05	*FZD4, VEGFA, KDR*
Wnt signaling pathway	4	226	5.41E-05	*FZD4, FZD5, NDP, LRP5*
Retinal blood vessel morphogenesis	2	6	5.88E-05	*FZD4, LRP5*
Positive regulation of transcription from RNA polymerase ii promoter in response to hypoxia	2	6	5.88E-05	*VEGFA, TP53*

**Table 2:** Top 10 enriched Kyoto Encyclopedia of Genes and Genomes (KEGG) pathways assigned to the text mining genes.

**PHPV-KEGG pathway**	**Genes in the query set**	**Total genes in the genome**	**Corrected *P*-value**	**Genes**
Pathways in cancer	9	368	1.24E-12	*IKBKG, FZD4, FZD5, VEGFA, TP53, TGFB2, SMAD2, LRP5, LAMB2*
Proteoglycans in cancer	7	141	5.54E-12	*FZD4, FZD5, VEGFA, TP53, TGFB2, SMAD2, KDR*
Hepatocellular carcinoma	6	90	3.16E-11	*FZD4, FZD5, TP53, TGFB2, SMAD2, LRP5*
Gastric cancer	6	89	3.94E-11	*FZD4, FZD5, TP53, TGFB2, SMAD2, LRP5*
Pancreatic cancer	5	62	8.60E-10	*IKBKG, VEGFA, TP53, TGFB2, SMAD2*
Human papillomavirus infection	6	179	1.39E-09	*IKBKG, FZD4, FZD5, VEGFA, TP53, LAMB2*
Hippo signaling pathway	5	81	2.42E-09	*FZD4, FZD5, TGFB2, NF2, SMAD2*
MAPK signaling pathway	5	192	1.66E-07	*IKBKG, VEGFA, TP53, TGFB2, KDR*
Wnt signaling pathway	4	69	1.96E-07	*FZD4, FZD5, TP53, LRP5*
Cell cycle	4	74	2.35E-07	*TP53, TGFB2, PCNA, SMAD2*
PI3K-Akt signaling pathway	5	222	2.49E-07	*IKBKG, VEGFA, TP53, LAMB2, KDR*
Breast cancer	4	87	3.77E-07	*FZD4, FZD5, TP53, LRP5*
Fluid shear stress and atherosclerosis	4	95	4.97E-07	*IKBKG, VEGFA, TP53, KDR*
Hepatitis B	4	127	1.49E-06	*IKBKG, TP53, TGFB2, PCNA*
Basal cell carcinoma	3	31	1.82E-06	*FZD4, FZD5, TP53*

### PPI Network Construction, Modular Analysis, and Key Genes Identification

**Figure 4:**
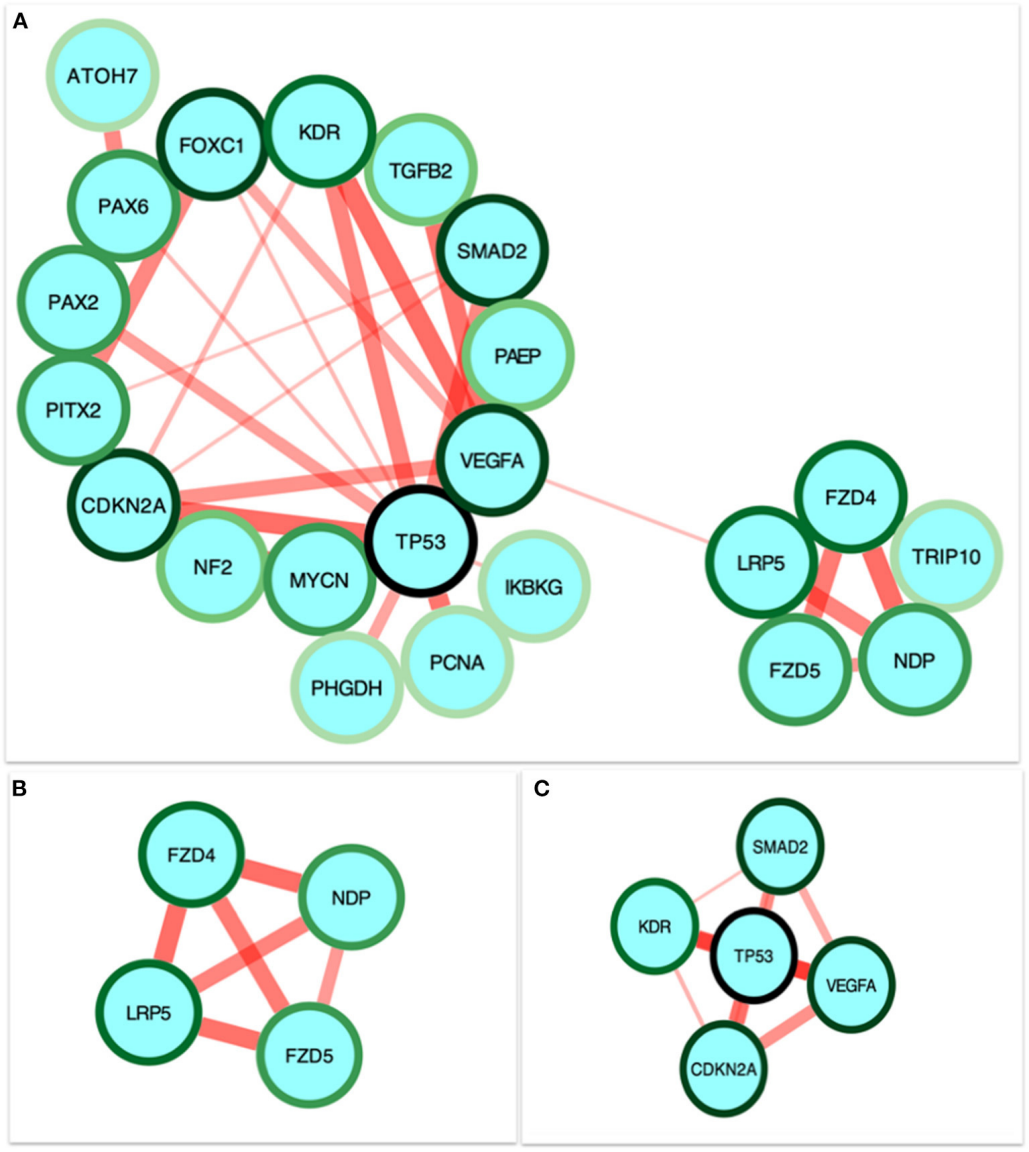
Identification and enrichment analysis of the text mining genes (TMGs). **(A)** The protein-protein interaction (PPI) network of the 35 target TMGs was visualized using Cytoscape. The gradience of the genes node border color reflects its role in the eye development process (a dark green node border represents the strongest degree of association and a light green node border represents the weakest degree of association). **(B,C)** The two modules were obtained from the PPI network using MCODE. **(B)** Module 1, the most significant module with four nodes. **(C)** Module 2 with five nodes.

**Figure 5:**
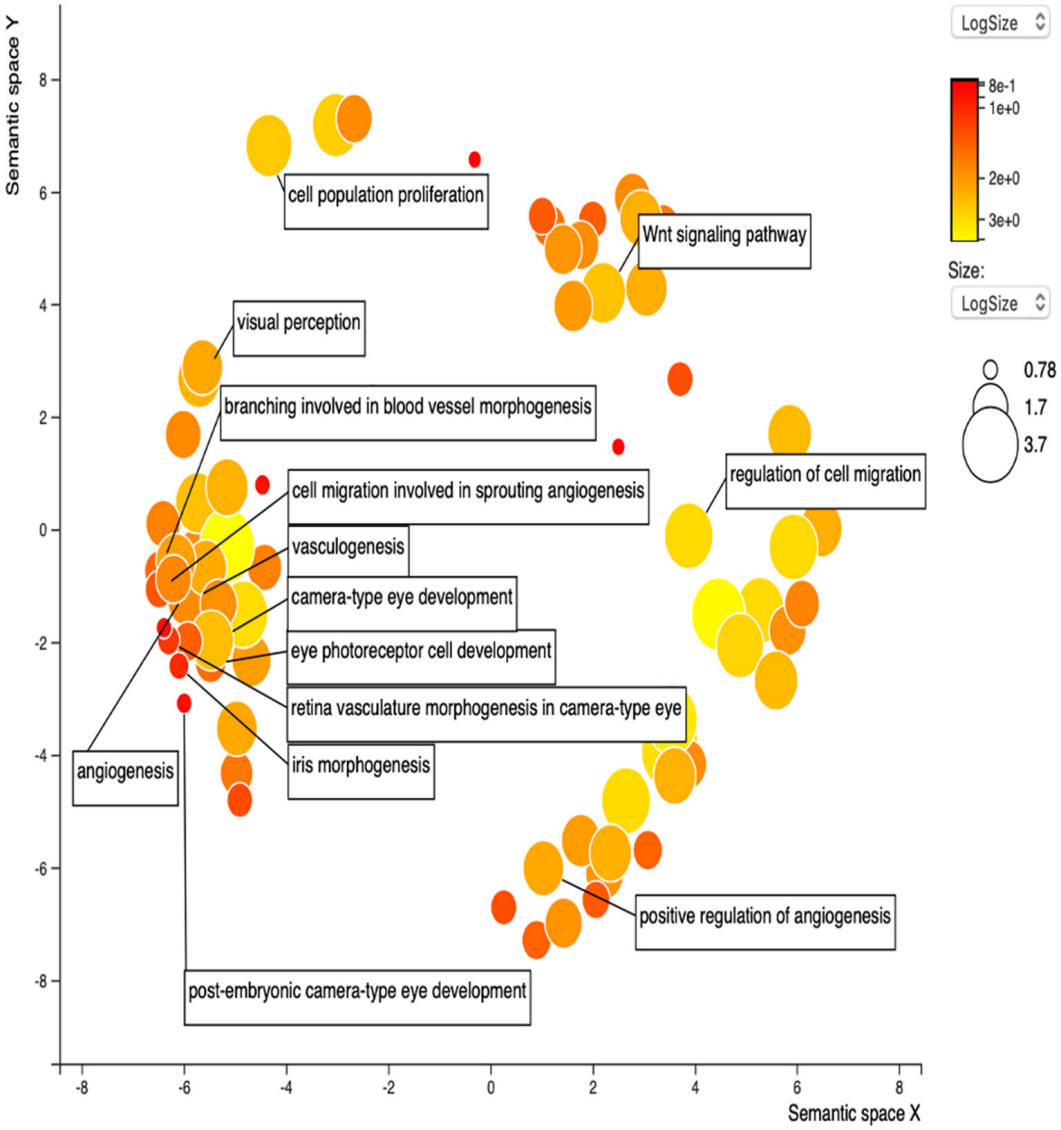
Gene Ontology terms of the 14 hub genes. The figure represents enriched GO terms related to eye morphogenesis and vasculature remodeling. DAVID and the REVIGO web server were employed to conduct functional and pathway enrichment analysis.

**Figure 6:**
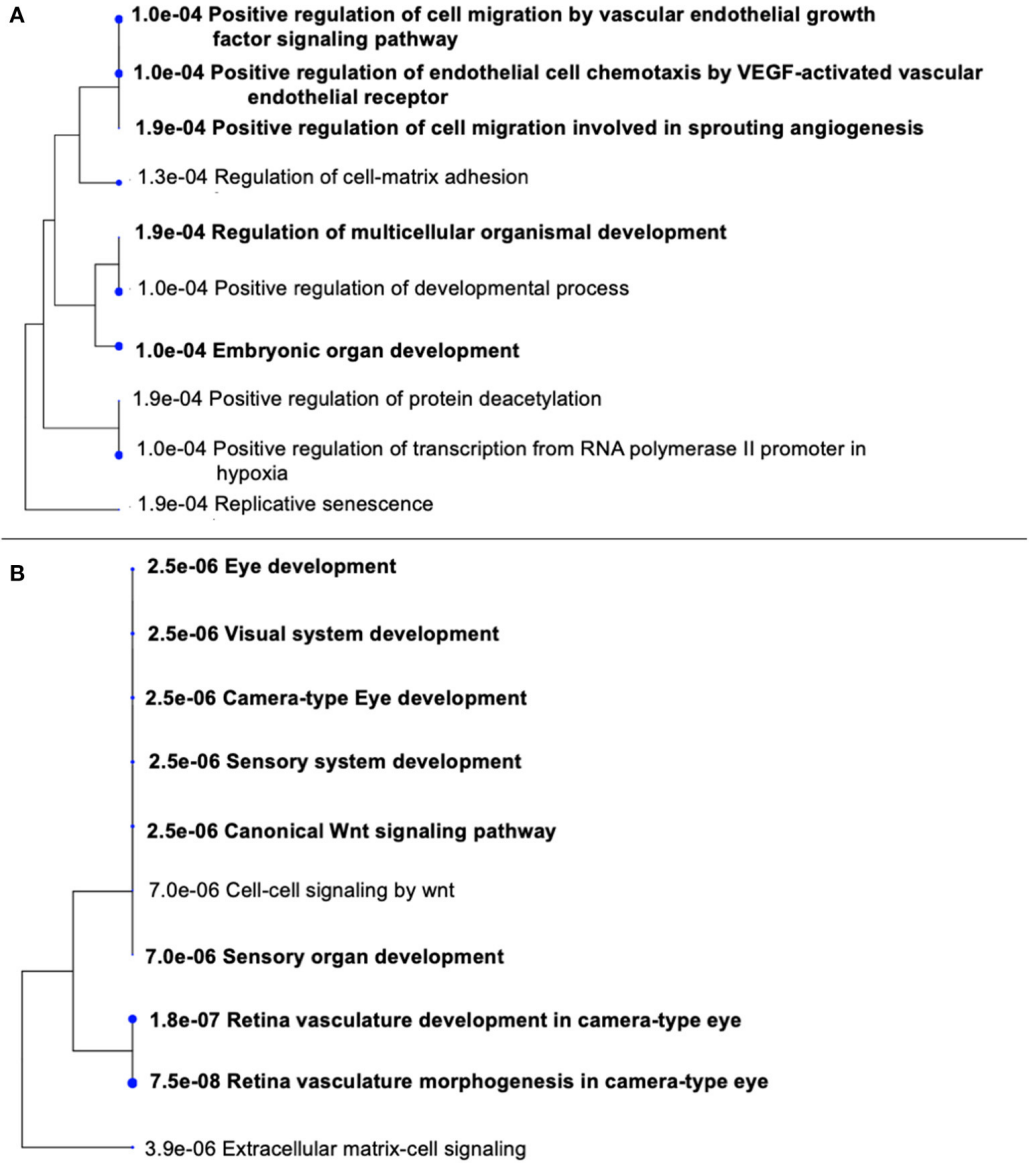
Gene Ontology (GO) terms in the three modules. **(A)** Significantly enriched GO terms in module 1. **(B)** Significantly enriched GO terms in module 2. The functional and pathway enrichment analyses in PHPV were related with high score (*P* > 0.005) based on a tree illustration created using the ShinyGo web server.

STRING was used to construct a PPI network for the 35 target genes with a high confidence score >0.900. There were 31 nodes and 42 edges in the network (Figure 4A). Using a cluster analysis of filtering nodes, 14 hub node genes were identified among 31 nodes (Table 3). The hub genes identified were *TP53, VEGFA, SMAD2, CDKN2A, FOXC, FZD4, LRP5, KDR, FZD5, PAX6, MYCN, NDP, PITX2*, and *PAX2*. The REVIGO analysis of the hub genes revealed five clusters based on GO similarity which were primarily related to eye development, Wnt signaling pathway, cell proliferation, regulation of cell migration, and regulation of angiogenesis (Figure 5). The modular analysis performed using MCODE yielded two modules. The PPI network relies on a total of 9 genes, as module 1 (*FZD4, FZD5, LRP5*, and *NDP)* contained 4 genes with 10 edges and module 2 (*TP53, KDR, VEGFA, CDK2NA*, and *SMAD2*) contained 5 genes with 6 edges (Figures 4B,C). According to the pathway enrichment analysis using KEGG and the ShinyGo platform, the genes in module 1 were associated with VEGF signaling pathway, regulation of execution process of apoptosis, and cell migration involved in sprouting angiogenesis. The module 2 genes were significantly associated with eye development, retinal vasculature development, and Wnt signaling pathway (Figure 6). Overall, the enrichment analysis revealed that these genes were substantially enriched in cell proliferation, anatomical structure morphogenesis, and regulation of developmental process which play a crucial role in vasculature formation of the lens causing PHPV.

**Table 3:** Hub node genes in the protein-protein interaction network identified with a filtering node degree ≥2.

**Genes**	**Degree**	**MCODE cluster**	**MCODE node status**	**MCODE SCORE**
*TP53*	12	Clustered	Module 1	4
*VEGFA*	9	Clustered	Module 1	4
*SMAD2*	7	Seed	Module 1	4
*CDKN2A*	6	Clustered	Module 1	4
*FOXC1*	5	Unclustered	–	1
*FZD4*	4	Clustered	Module 2	3
*LRP5*	4	Clustered	Module 2	3
*KDR*	4	Clustered	Module 1	4
*FZD5*	3	Clustered	Module 2	3
*PAX6*	3	Unclustered	–	2
*MYCN*	3	Unclustered	–	3
*NDP*	3	Seed	Module 2	3
*PITX2*	3	Unclustered	–	2
*PAX2*	3	Unclustered	–	2

### Drug-Gene Interaction Analysis of Core Genes

In the drug-gene interaction study, we selected 14 hub genes as potential drug targets (Table 4). Overall, 7 of the 14 are potential gene targets and 26 FDA-approved drugs are expected to have drug-gene interactions. FOXC*1, FZD4, LRP5, FZD5, PAX6, NDP*, and *PITX2* were the exceptions. The major interactions among drugs, genes, and pathways are depicted in Table 4. Table 5 represents the chemical structure and formula of the identified drugs.

**Table 4:** Details of the 26 Food and Drug Administration (FDA)-approved drugs that potentially target 7 of the 14 hub genes.

**Drug**	**Gene**	**Interaction**	**Interaction score**	**Drug class**	**Approved**	**Reference (PubMed ID)**
Ranibizumab	*VEGFA*	Inhibitor	6.51	Antineoplastic agents, ocular vascular disorder agents	Yes	18046235, 18054637
Pegaptanib Sodium	*VEGFA*	Antagonist	2.31	Antagonist agent vascular endothelial growth factor (VEGF)	Yes	23953100
Aflibercept	*VEGFA*	Antibody, binder, inhibitor	1.89	Antagonist agent vascular endothelial growth factor-A (VEGF-A) and placental growth factor (PLGF)	Yes	22813448, 20124951
Minocycline	*VEGFA*	Inhibitor	0.28	Antibiotic agent	Yes	11875741, 16224178
Carvedilol	*VEGFA*	Other/unknown	0.18	Beta blocker agent	Yes	15071347, 15942707, 15732037
Dinutuximab	*MYCN*	Other/unknown	6.31	Antineoplastic agents	Yes	–
Sonidegib	*MYCN*	Other/unknown	2.52	Antineoplastic agents	Yes	24651015
Amifostine	*SMAD2*	Unknown	2.24	Chemo protectant, antineoplastic adjunct, or cytoprotective agent	Yes	–
Hydrocortisone	*SMAD2*	Unknown	0.3	Corticosteroids	Yes	26343583, 27488531, 22983396
Bleomycin	*SMAD2*	Unknown	0.57	Antitumor antibiotic	Yes	–
Tretinoin	*SMAD2*	Unknown	0.26	Anticancer drugs	Yes	–
Progesterone	*PAX2*	Unknown	1.48	Antimineralocorticoid; neurosteroid	Yes	11850818
Vandetanib	*KDR*	Inhibitor	0.0	Tyrosine kinase inhibitors	Yes	26578684, 20124951
Ramucirumab	*KDR*	Inhibitor, antagonist, antibody	0.63	Binds to the vascular endothelial growth factor receptor-2	Yes	20048182, 2182794
Lenvatinib	*KDR*	Inhibitor		Kinase inhibitors, antineoplastic agent	Yes	17943726
Sunitinib	*KDR*	Inhibitor	0.25	Kinase inhibitors, antineoplastic agent	Yes	27149458, 20142593, 25639617
Sorafenib	*KDR*	Antagonist, inhibitor		Kinase inhibitors, antineoplastic agent	Yes	16824050, 16418310, 26344591
Axinib	*KDR*	Unknown	0.32	Kinase inhibitors, antineoplastic agent	Yes	–
Regorafenib	*KDR*	Inhibitor	0.14	Kinase inhibitors, antineoplastic agent	Yes	27004155
Sorafenib	*KDR*	Antagonist, inhibitor	0.12	Kinase inhibitors, antineoplastic agent	Yes	16824050, 16418310, 26344591
Abemaciclib	*CDKN2A*	Unknown	0.92	Antineoplastic agent	Yes	27217383, 26183925
Palbociclib	*CDKN2A*	Unknown	0.88	Antineoplastic agent	Yes	26715889, 21278246, 22711607
Alectinib	*CDKN2A*	Unknown	0.65	Antineoplastic agent	Yes	–
Zinc Chloride	*TP53*	Chaperone	0	Immune system functioning agent	Yes	17327663, 29843463
Trifluridine	*TP53*	Unknown	0.07	Ophthalmological antiviral agent	Yes	25700705
Bortezomib	*TP53*	Inhibitor	0	Anticancer drug	Yes	28679691

**Table 5:** Chemical structure of the potential drugs that target the seven candidate genes.

**Drug**	**Gene target**	**Chemical structure**	**Chemical formula/protein formula**
Pegaptanib sodium	*VEGFA*	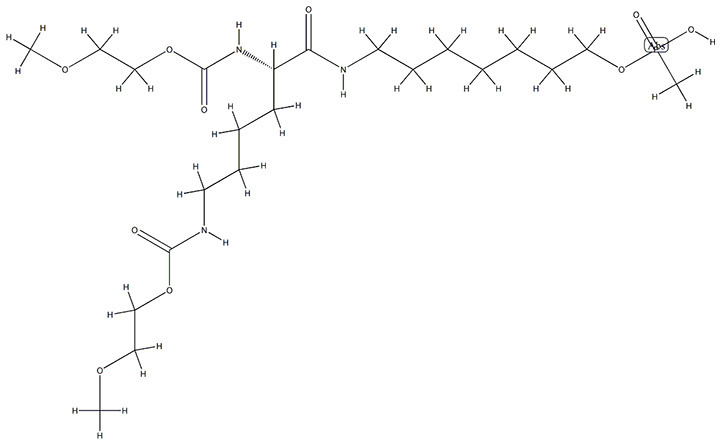	C_22_H_44_N_3_O_10_P
Minocycline	*VEGFA*	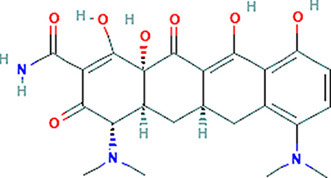	C_23_H_27_N_3_O_7_
Carvedilol	*VEGFA*	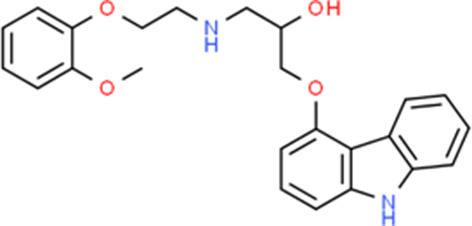	C_24_H_26_N_2_O_4_
Sonidegib	*MYCN*	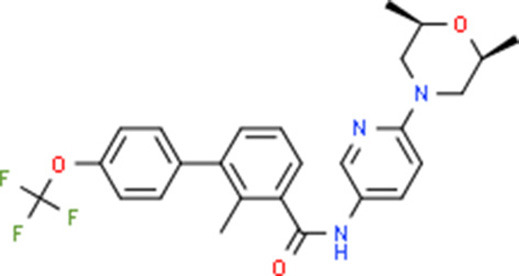	C_26_H_26_F_3_N_3_O_3_
Amifostine	*SMAD2*	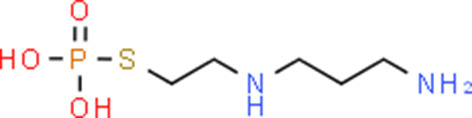	C_5_H_15_N_2_O_3_PS
Hydrocortisone	*SMAD2*	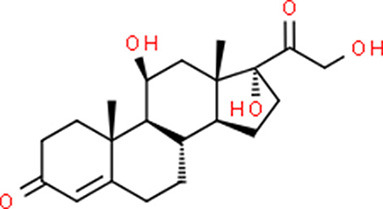	C_21_H_30_O_5_
Bleomycin	*SMAD2*	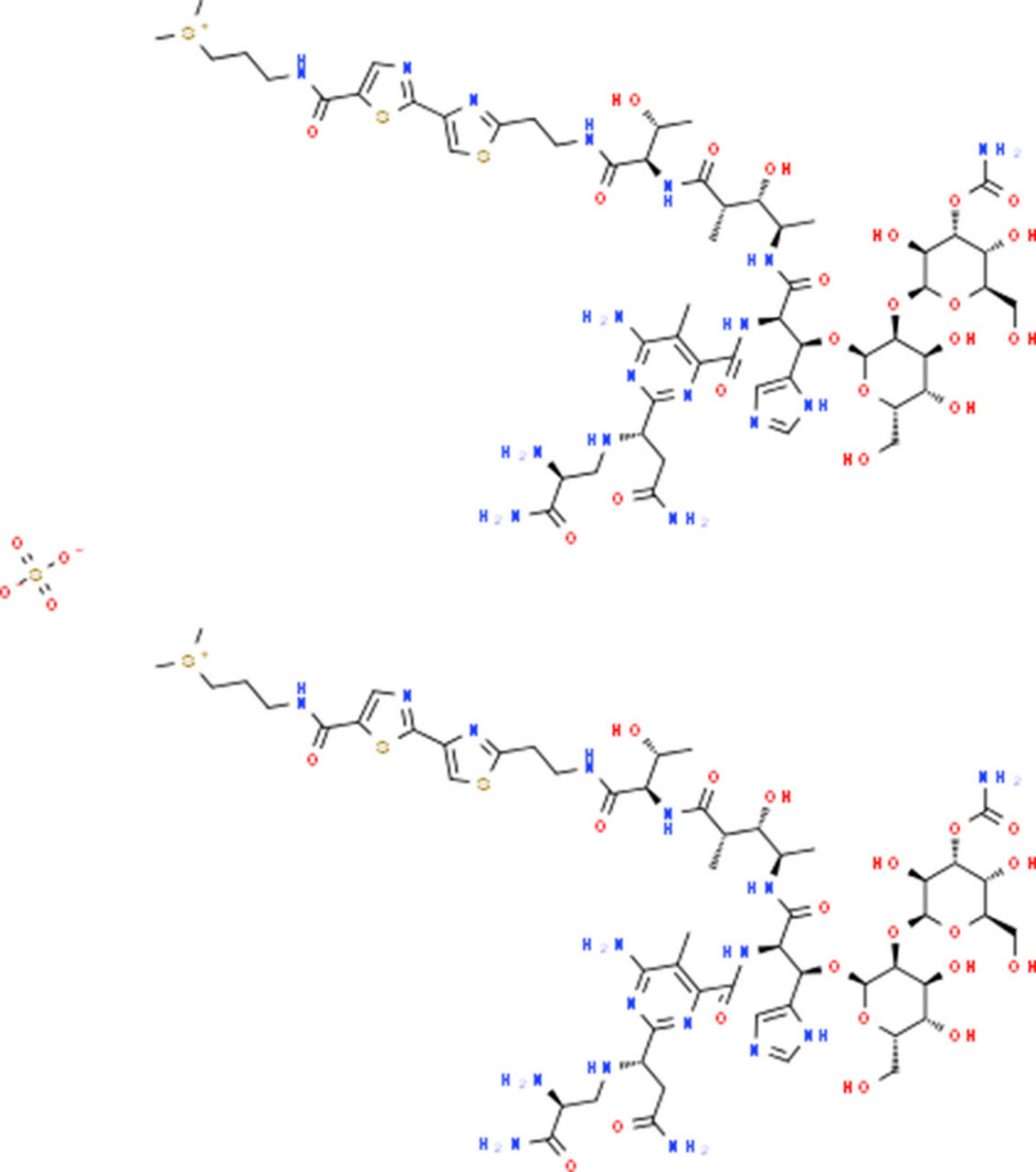	C_110_H_168_N_34_O_46_S_7_
Tretinoin	*SMAD2*	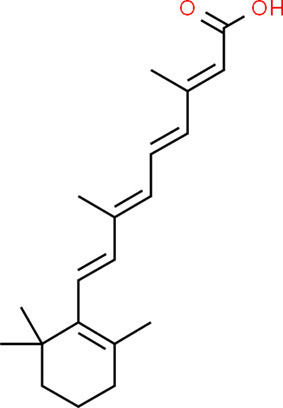	C_20_H_28_O_2_
Progesterone	*PAX2*	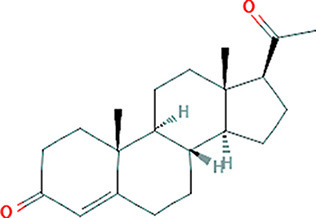	C_21_H_30_O_2_
Vandetanib	*KDR*	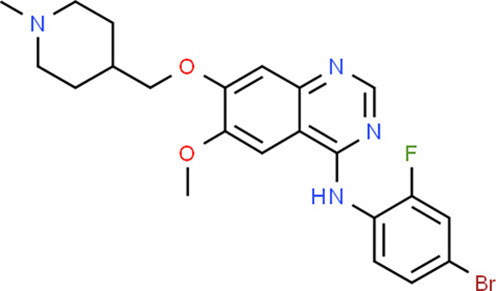	C_22_H_24_BrFN_4_O_2_
Lenvatinib	*KDR*	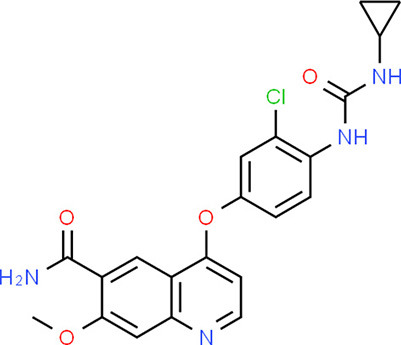	_21_H_19_ClN_4_O_4_
Sunitinib	*KDR*	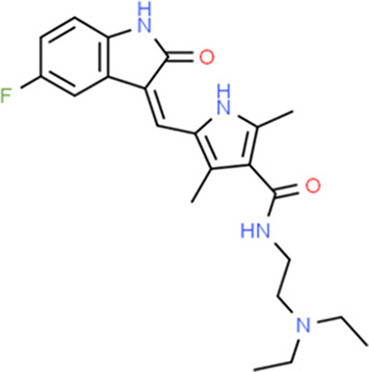	C_22_H_27_FN_4_O_2_
Sorafenib	*KDR*	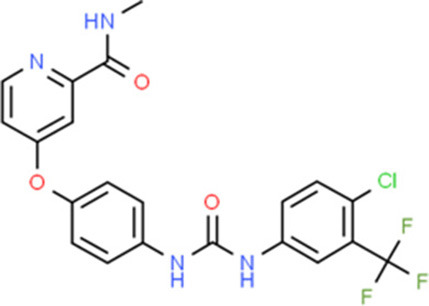	C_21_H_16_ClF_3_N_4_O_3_
Axinib	*KDR*	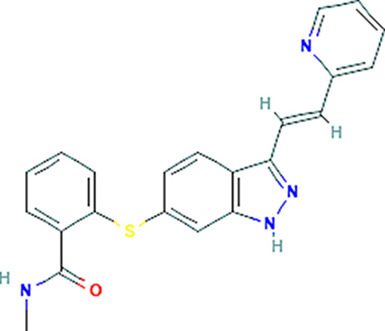	C_22_H_18_N_4_OS
Regorafenib	*KDR*	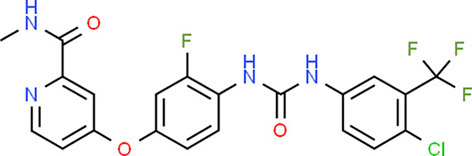	C_21_H_15_ClF_4_NO_3_
Sorafenib	*KDR*	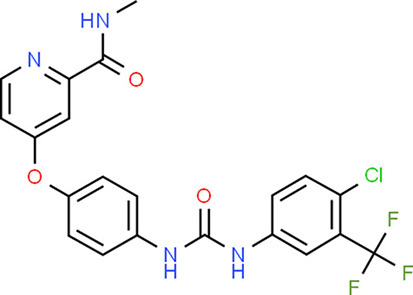	C_21_H_16_ClF_3_N_4_O_3_
Abemaciclib	*CDKN2A*	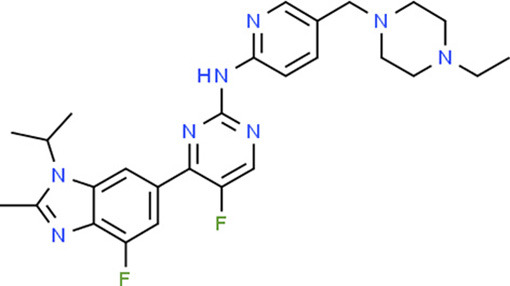	C_27_H_32_F_2_N_8_
Palbociclib	*CDKN2A*	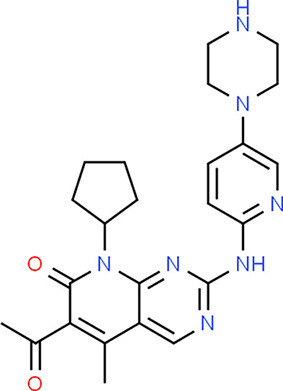	C_24_H_29_N_7_O_2_
Alectinib	*CDKN2A*	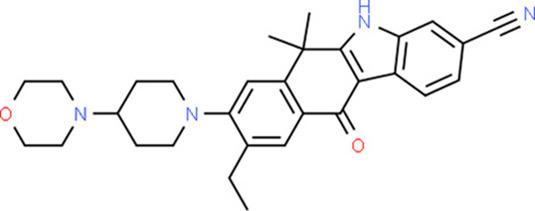	C_30_H_34_N_4_O_2_
Zinc chloride	*TP53*	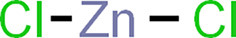	Cl_2_Zn
Trifluridine	*TP53*	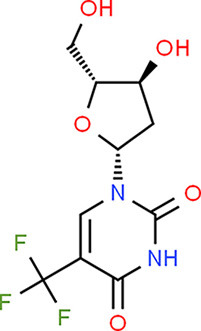	C_10_H_11_F_3_N_2_O_5_
Bortezomib	*TP53*	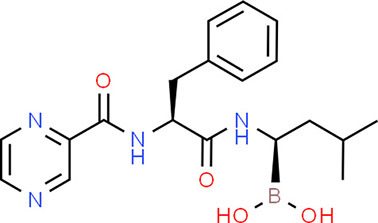	C_19_H_25_BN_4_O_4_
Ranibizumab	*VEGFA*	Monoclonal antibody	C_2158_H_3282_N_562_O_681_S_12_
Aflibercept	*VEGFA*	Monoclonal antibody	C_4318_H_6788_N_1164_S_32_
Dinutuximab	*MYCN*	Monoclonal antibody	C_6422_H_9982_N_1722_O_2008_S_48_
Ramucirumab	*KDR*	Monoclonal antibody	C_6374_H_9864_N_1692_O_1996_S_46_

## Discussion

**Figure 7:**
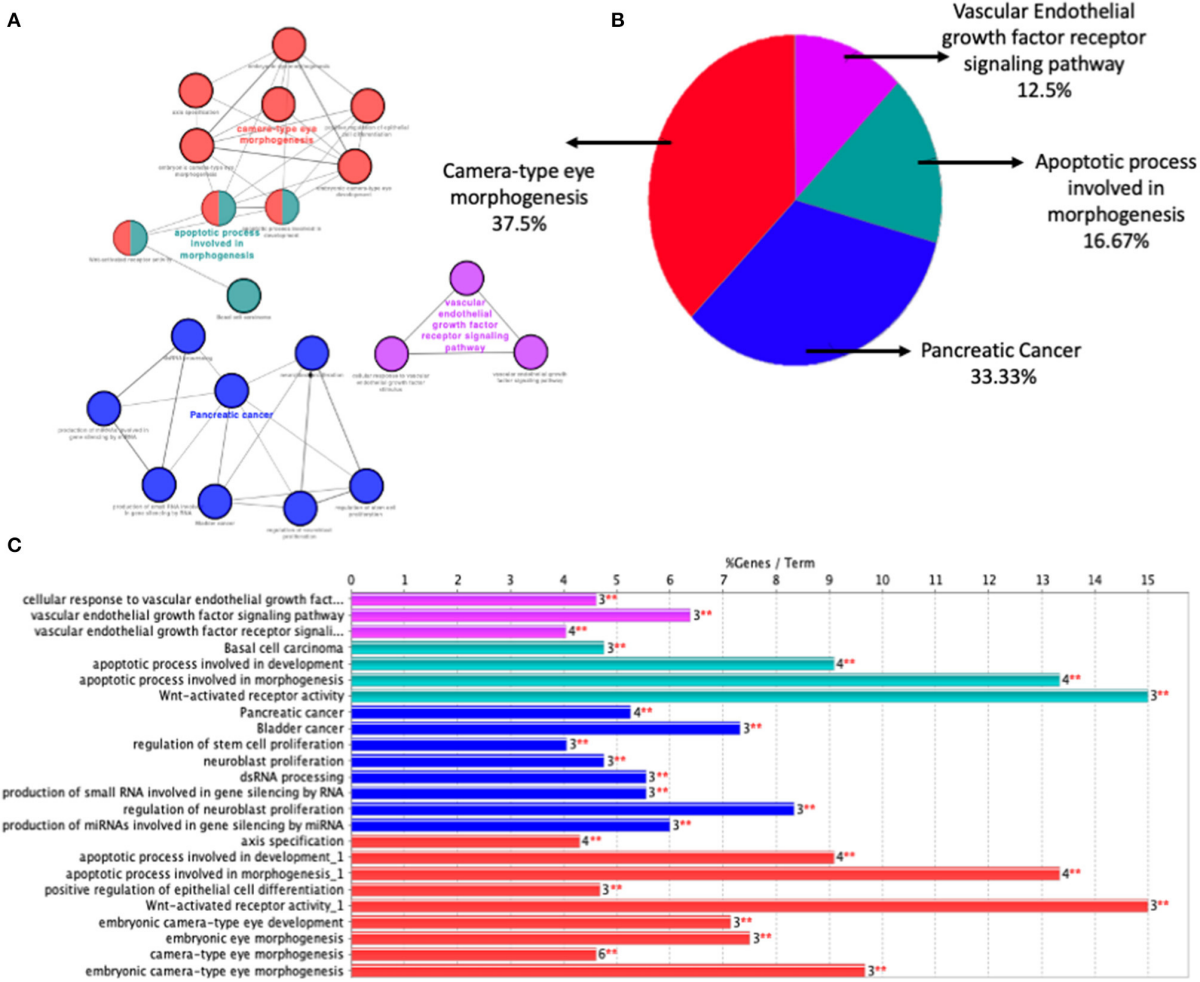
Function analysis of the 14 core genes in module 1 and module 2. **(A)** Functions and pathways of the core genes were visualized using ClueGO. **(B)** Distribution of the functions and pathways among the core genes. **(C)** KEGG pathways and enriched GO terms, colors are assigned to each pathway. Corrected *P* < 0.01 was considered statistically significant.

PHPV is a disease that leads to blindness or severe vision loss, although there are currently few therapeutic choices (24, 25). On the other hand, PHPV patients are more likely to develop cataracts and closed-angle glaucoma early on in life. Terminal glaucoma, uveitis, retinal detachment, and intra-ocular hemorrhage can be inevitable for these patients (35). As a consequence, the molecular mechanisms that contribute to PHPV must be established. Our analysis discloses that the molecular mechanism of PHPV overlaps with various other signaling pathways contributing to a broader range of therapeutic targets and prognostic biomarkers. The present paper reports 35 genes that might be involved in the development of the eye's vasculature process in the PHPV condition. The enriched GO and BP terms assigned to these genes were associated mainly with extracellular matrix-cell signaling, multicellular organism development, regulation of transcription by RNA polymerase ii, Norrin signaling pathway, and retina vasculature morphogenesis in camera-type eyes. The PPI network and enrichment analysis identified 14 hub genes, *TP53, VEGFA, SMAD2, CDKN2A, FOXC, FZD4, LRP5, KDR, FZD5, PAX6, MYCN, NDP, PITX2*, and *PAX2* that were involved in camera-type eye morphogenesis, pancreatic cancer, the apoptotic process involved in morphogenesis, and the VEGF receptor signaling pathway (Figure 7). The functional analysis and pathways of the key genes in module 1 and module 2 illustrated using ClueGO are displayed in Figure 7A. Figure 7B displays the distribution of functions and pathways among core genes, while Figure 7C reveals KEGG pathways and enriched GO terms, with colors allocated to each pathway.

Based on the evaluations, four genes such as *FZD4, LRP5, FZD5, NDP* were involved in the process of eye development, retina vasculature development, retinal blood vessel morphogenesis, and the Wnt signaling pathway [Figure 6B; (43, 44)]. The architecture of the retinal vasculature is dependent on highly organized signaling between various cell types of retina, combining internal metabolic conditions with external influences such as oxygen and nutrient supply. In various organs, including the eye, the Wnt signaling pathway is essential for vascular morphogenesis. During eye development, and in vascular eye disorders, Wnt ligands and receptors are key regulators of ocular angiogenesis and also control the development of structured layers of vasculature in retinas as well as the regression of hyaloid vessels (45). FEVR (an inherited disease in which the peripheral retina is hypovascularized to varying degrees) has been attributed to mutations in Wnt pathway components *FZD4, LRP5*, and the secreted cysteine-knot protein Norrin (46, 47). Norrin is a non-Wnt ligand with a high affinity *FZD4* receptor located in the retina and activates the Wnt/β-catenin pathway. Norrie disease, retinopathy of prematurity, and Coats disease are vascular retinopathies caused by defects in the Norrin gene (48). In humans, mutations in *NDP* and *FZD4* have been identified in a limited number of unilateral and bilateral PHPV patients (14, 49–51). *ATOH7* mutation (N46H-homozygous) in a family of autosomal recessive PHPV disease traits linked to 10q21 has been identified (52). These variations include deletions, insertions, and missense and nonsense mutations. However, individuals with X-linked FEVR, autosomal dominant FEVR, retinopathy of prematurity, and Norrie disease have also been reported to have mutations in *NDP* and *FZD4* genes (53). According to the GO analysis, five genes *TP53, VEGFA, SMAD2, CDKN2A*, and KDR (Figure 6A) are involved in the process of regulation of cell migration by the VEGF signaling pathway, angiogenesis, regulation of muscle cell apoptotic process, and embryonic organ development process. Apoptosis is another crucial process in eye development involving extensive programmed cell death associated with morphogenesis (54).

Previous research on transgenic mice models supports our *in silico* analysis of PHPV to validate the function of these hub genes in hyaloid vasculature regression such as knockouts of the *Arf* tumor suppressor gene (23, 55, 56), *p53* (21, 57), and *Frizzled-5* (57) which were associated with PHPV-like phenotypes in mouse models. During mouse eye development, the *arf* tumor suppressor gene promoted hyaloid vasculature regression and its deficiency may cause a retrolental membrane with persistent hyaloid vessels (9, 23). In *Atoh7* knockout mice, hyaloid vessels persist in the vitreous and proliferate to supply the retina which lacks intrinsic vasculature (58, 59). The ephirin-A5 family of receptor tyrosine has been demonstrated to be significant in the regression of the primary vitreous in mouse models (60). Furthermore, in mice lacking *LRP5*, a Wnt receptor displayed hyaloid vasculature that lasted throughout their lives (61, 62). Given the correlation between transgenic mouse PHPV phenotypes and the hub genes in human congenital defects affecting the eye morphogenesis or retinal vasculature and molecular signaling pathways in module 1 and module 2, it suggests that the pathogenesis of PHPV is regulated by genes in modules 1 and 2.

Twenty-six drugs identified by the drug-gene interaction analysis were classified as anti-neoplastic agents, ocular vascular disorder agents, kinase inhibitors, immune system functioning agents, or corticosteroids. Among them, four potential drugs such as Ranibizumab, Dinutuximab, Pegaptanib Sodium, and Sonidegib were identified based on their high drug-gene interaction score (Table 4). Ranibizumab is a recombinant humanized monoclonal antibody fragment that binds to human vascular endothelial growth factor A (VEGF-A) and thereby prevents it from binding to its receptor and blocking the development of new blood vessels (63). Pegaptanib Sodium is an anti-angiogenic drug used to treat neovascular diseases. It specifically binds to the 165 isoform of VEGF, a protein that is involved in angiogenesis and increased blood vessel leakage (64). Ranibizumab and Pegaptanib Sodium are typically used to treat wet age-related macular degeneration, a type of eye disease (61, 62, 65, 66). They are also used to treat macular edema after retinal vein occlusion, diabetic macular edema, and diabetic retinopathy. Dinutuximab is a GD2-binding human/mouse chimeric monoclonal antibody. It has been proven that the action of pro and anti-angiogenic factors regulates angiogenesis in the development of new capillaries from a pre-existing capillary network (67). Dinutuximab binds to GD2 on the cell surface and induces GD2 expressing cells to lyse by antibody-dependent cell-mediated cytotoxicity and complement-dependent cytotoxicity (68, 69). Sonidegib is an anticancer drug that inhibits the hedgehog (Hh) pathway which is involved in cell differentiation, tissue polarity, and stem cell maintenance during embryonic growth. Hh is essential for the development of the hyaloid loop on the lens's ventral surface by promoting VEGF-mediated angiogenesis. In a zebrafish model, the loss of Hh signaling induced excess sprouting of blood vessels in the dorsal eye and impaired the growth of blood vessels in the ventral eye (70). Regulation of the Hh signaling pathway has been associated with the growth and progression of cancers such as basal cell carcinoma, medulloblastoma (71), and periocular basal cell carcinoma (72).

In PHPV, the ocular fetal vasculature does not go through normal developmental regression. The reasons could be due to presumed loss of apoptosis in PHPV; these natural apoptotic pathways could be pathologically disrupted (22, 73). Apoptosis is a process of cell death that is regulated by a number of gene families. In mice models, the macrophage has been established as a key mediator in studies examining the mechanisms of regression (74, 75). *In silico* drug-gene analysis using the hub genes of PHPV revealed high interaction with anticancer compounds in the present study. It is understood that vascular quiescence can be regulated by a combination of pro and anti-angiogenic factors. Previous reports have demonstrated that the equilibrium of angiogenic factors such as VEGF and placental growth factor is crucial in vascular regression and mice lacking angiopoietin 2 which regulates angiogenesis by binding to the Tie2 receptor, maintaining fetal vessels in the eyes (18, 76). In addition, the identified drugs can be used for pharmacological screening in mice and zebrafish models to identify compounds affecting vasculature development that could be of therapeutic importance. The results of the study could lead to a better understanding of the potential molecular pathway and possible hyaloid vasculature mechanism, as well as the development of novel therapeutics to prevent or cure this blinding disease, PHPV. Since the current paper focuses on the appropriate path for understanding molecular pathways and therapeutic options for PHPV through *in silico* analysis, further experimental analysis using animal models is highly recommended to confirm the significance of the candidate genes and pro- and antiangiogenic factors in hyaloid vasculature development and physiology. This continues to be a limitation of the study.

## Conclusion and Future Perspectives

To conclude, no specific candidate genes, molecular pathways, or drug targets have been associated with PHPV until now. *TP53, VEGFA, SMAD2, CDKN2A, FOXC, FZD4, LRP5, KDR, FZD5, PAX6, MYCN, NDP, PITX2*, and *PAX2* were identified for the first time as hub genes using *in silico* tools that may be involved in the development of retinal vasculature and dysfunction of these genes, leading to PHPV. These genes appear to be predominantly associated with functions related to eye morphogenesis, cancer, apoptosis, and VEGF receptor signaling pathways. Previous reports in knockout *TP53, VEGFA, FZD4*, and *NDP* transgenic mouse models confirmed the failure of regression of hyaloid vessels and abnormalities in the retinal vasculature. In the future, these *in silico* analyses will be validated by mutation screening of the hub genes in PHPV patients in order to identify pathogenic variants and gene product expressivity. Clearly, more research is warranted on animals and in human patients as the phenotypic differences will differ from individual to individual based on the expressivity of the gene product. In addition, we identified four genes that may be potential drug targets. Precision medicine for a fetal ocular condition like PHPV presents new challenges but with a possibility. Since PHPV is a rare and often autosomal recessive condition, the present paper is useful when there is little pathological knowledge about the disease or where there is substantial pathway heterogeneity, underlying the clinical phenotype. As a result, a combination of therapeutic methods such as surgical intervention and candidate gene identification may be used not only to analyze biological pathways unique to specific cases, but also to propose potential drug combinations based on gene products annotated to the disease associated with PHPV. This research sheds light on the potential of personalized intervention in the treatment of PHPV indicating a substantial advancement in management strategy.

## Data Availability Statement

The raw data supporting the conclusions of this article will be made available by the authors, without undue reservation.

## Ethics Statement

Written informed consent was obtained from the individual(s), and minor(s)' legal guardian/next of kin, for the publication of any potentially identifiable images or data included in this article.

## Author Contributions

DT, CK, and DB conceived, designed the study, and wrote the manuscript. All authors contributed and wrote parts of the manuscript and approved the submitted version.

## Conflict of Interest

The authors declare that the research was conducted in the absence of any commercial or financial relationships that could be construed as a potential conflict of interest.

## Publisher's Note

All claims expressed in this article are solely those of the authors and do not necessarily represent those of their affiliated organizations, or those of the publisher, the editors and the reviewers. Any product that may be evaluated in this article, or claim that may be made by its manufacturer, is not guaranteed or endorsed by the publisher.
